# Theoretical report: reflections and considerations for authors, reviewers, and editors

**DOI:** 10.11606/s1518-8787.2022056003766

**Published:** 2022-04-13

**Authors:** Breno Augusto Bormann de Souza, Érika Fernandes Tritany, Cláudio José Struchiner

**Affiliations:** I Escola Nacional de Saúde Pública Departamento de Epidemiologia em Saúde Pública Programa de Doutorado em Epidemiologia em Saúde Pública Rio de Janeiro RJ Brasil Escola Nacional de Saúde Pública. Departamento de Epidemiologia em Saúde Pública. Programa de Doutorado em Epidemiologia em Saúde Pública. Rio de Janeiro, RJ, Brasil; II Universidade de Pernambuco Escola Superior de Educação Física Recife PE Brasil Universidade de Pernambuco. Escola Superior de Educação Física. Recife, PE, Brasil; III Universidade Federal do Rio de Janeiro Faculdade de Medicina Macaé RJ Brasil Universidade Federal do Rio de Janeiro. Faculdade de Medicina. Macaé, RJ, Brasil; IV Universidade Federal do Rio Grande do Norte Programa de Doutorado em Saúde Coletiva Natal RN Brasil Universidade Federal do Rio Grande do Norte. Programa de Doutorado em Saúde Coletiva. Natal, RN, Brasil; V Fundação Getúlio Vargas Departamento de Matemática Aplicada Rio de Janeiro RJ Brasil Fundação Getúlio Vargas. Departamento de Matemática Aplicada. Rio de Janeiro, RJ, Brasil; VI Universidade Estadual do Rio de Janeiro Instituto de Medicina Social Rio de Janeiro RJ Brasil Universidade Estadual do Rio de Janeiro. Instituto de Medicina Social. Rio de Janeiro, RJ, Brasil

**Keywords:** Epidemiologic Studies, Grounded Theory, Measurements, Methods and Theories, Publications

## Abstract

Epidemiological studies focused on public health have currently shown significant limitations regarding in-depth theoretical reports, overvaluing methodological aspects. The lack of theoretical explanation affects both the quality and reproducibility of studies. This study therefore reflected on the importance of in-depth theoretical reports considering the theoretical foundation used by researchers in the main sections of the manuscript (title, abstract, introduction, methodology, results, discussion, and conclusion) based on a review of the scientific literature on the subject. We believe that this article can help understand the importance and the development of in-depth theoretical reports in scientific articles, contributing to assessments, interpretations, and criticisms of reviewers and editors regarding the explanation and reporting of theoretical foundation in manuscripts submitted to scientific journals.

## INTRODUCTION

Theory and methodology are considered essential to the scientific doing since they give rigor and quality to research. Although the literature describes the importance of combining these two elements, they are often applied unequally^1^. Reporting failures (theoretical or methodological) can also reduce the validity of findings and negatively affect the reproducibility of studies^2^.

In reports of epidemiological studies, methodological aspects are increasingly overlapping theoretical aspects. Pressure for productivity and demands for publications in high-impact journals often guide research leaders to methodological preferences, limiting the range of studies to be developed^3^.

Journals also reinforce this attitude by requiring specific instruments to measure potential methodological bias or reporting guidelines developed for specific designs (e.g., PRISMA, STROBE, CONSORT), which do not concern theoretical issues. Moreover, journals offer no guidelines for reporting theoretical foundation and authors have few instruments that help conduct an in-depth theoretical report.

All studies must be related to a theory and/or theoretical model that supports and guides the stages of the research^4^. The in-depth report of such theory should facilitate the study’s identification and guide both the research team and the reader. In epidemiological studies, the absence of citations and explanation of theories or theoretical models can affect the understanding of the study, limit critical evaluation, and compromise quality and reproducibility^2,5,6^ since reproducibility does not depend on methodological quality alone, but on the combination between methodological, theoretical, and reporting qualities^2^.

Cabrera^1^ considers that rigorous scientific communication should quote the adopted theory and describe its variables and constructs, explain how the theoretical foundation guided the methodology and its analyses, and, finally, discuss the conclusions or thematic issues addressed based on the explanatory components of the theory or descriptions of the theoretical models. A theoretical report should not focus on mechanically and exhaustively repeating the theoretical framework, but on understanding its importance in all stages of research and manuscript.

Thus, quoting “... this study was based on the theory/theoretical model x” in each part of the text is insufficient. The author must explain the theory’s contributions to each section of the article according to its function in the text and the possible effects of the study on the Theory/Model. Our study therefore reflected about the importance of an in-depth theoretical report in each section of a scientific article.

## TITLE

The title should briefly reflect the essence of the manuscript and its novelty and relevance to science^7^, but the literature is yet to agree on an ideal title size^8,9^. It should summarize the main idea of the manuscript in a simple and stylish way, identifying key variables, theories adopted, and the relationship between them^10^while being sufficiently informative, descriptive, and precise to attract interest and favor the study’s identification^11^.

Studies that do not have the theory/theoretical model in the title might not be selected in literature reviews and bibliographic searches on the theory and its use. Title-reading is often suggested as an initial stage of bibliographic search and selection. Titles that do not mention the adopted theory could thus hinder bibliographic findings, decreasing the range of literature reviews^12^.

Therefore, mentioning the theory in the title can help researchers identify the article and promptly show readers the theoretical approach adopted. After all, determining the factor that makes the article unique in the field is important, and emphasizing it on title could stimulate interest and the potential access of readers^11^.

## ABSTRACT

The abstract seeks to help readers select appropriate articles more quickly, allowing more accurate searches and facilitating peer review^13^. They can be “structured” or “unstructured”, in which content can be the same, but in different presentation formats^14^. Many journals recommend structured abstracts since they have shown to be more effective and systematic^15^.

Abstracts offer reviewers an immediate and general meaning of the study, helping them structure the analysis and review the article^15^. However, despite their advantages^13^, abstracts often have missing information which are only present in the text^16^, especially information related to the theoretical foundation and its contribution in sections of the manuscript^17^.

An abstract must contain preliminary information about the theoretical framework, explaining how the variables were evaluated, the main findings, potential limitations, and conclusions related to the theory/theoretical model adopted, even if simplified^10,18^. Its synthetic explanation of the used methodology, results obtained, limitations identified, and conclusions – sections already commonly reported in studies and abstracts – considers the challenges and potentialities of the theory/model used, its contribution to the study, and how the research could strengthen or question it. This practice provides a coherent and cohesive report, increasing the article’s chance of selection, access, and evaluation.

## INTRODUCTION

The introduction should briefly explain the study’s subject according to the literature and the theoretical foundation adopted^19^, presenting current knowledge, recent insights and developments on the subject, and possible gaps to justify theoretical and methodological choices^20^. This section gives authors greater freedom to explain the study’s context and basis broadly and descriptively.

However, it is often described succinctly, seeking to convince the reader of the value of its product^21^. This attitude – popularly known as “selling fish” in Brazil – should be combated, since it often overvalues methodological aspects from the pressure for increased scientific productivity over quality^22,23^.

The introduction should justify the theoretical methodological choices and present questions to be answered in the text, explaining and describing to the reader: the theoretical foundation; the reference used; their scientific appreciation or criticism; parallel theories or gaps in the literature; and reasons that guided their choice, based on hypotheses about how the theory/theoretical model can contribute to the studied phenomenon.

Moreover, this initial presentation can include more than consecrated theories and great canons of literature^5^. Many researchers innovate regarding causal relationships but lack literature to support their hypotheses, thus creating theoretical models based on the free connection between different themes and/or previous scientific findings. In this case, they must explain the theoretical model created, their variables and inclusion or exclusion criteria, their interrelations, and possibilities of defending the model as innovative for the theme.

## METHODOLOGY

All knowledge carries theory, and all methods are guided by theories^24^. Although the methodology section commonly presents only the research/intervention methods used, it should also report on the theoretical foundation that supports the methodological path, explaining how the theory/theoretical model guided the method and how the variables were classified and interrelated.

A profound explanation of the theoretical-methodological path adopted includes justifying the exclusion and inclusion of variables in the analyses and the presence of bias. Authors should not only quote the chosen method – disconnected from the theoretical framework and the evidence on the subject – but explain, in depth, each point of importance for the analyses and if they correspond with the theoretical foundation.

We believe that the choice of variables is guided by the theory/theoretical model (existing, adapted or new, created by researchers) and not by arbitrary decisions such as ease/difficulty in obtaining data or even preferences of researchers. Authors should justify if they cannot obtain information on important variables.

Moreover, a graphic presentation of the theoretical model can better explain the variables involved and their relations^2^. Every theoretical model is a simplification of reality and so is its graphic representation^25^. However, researchers and readers might have difficulty visualizing the chosen theoretical model among the large textual volume produced, detailed descriptions of phenomena, and criticisms and considerations^6^.

The graphic representation of the theoretical model is essential for the communication of the research since it facilitates the visualization of the variables of interest and their interrelations, promotes significant learning about the studied phenomenon^2^, continuously improves the theoretical model by reviewing variables and observing relationships not initially considered, and helps create the model of analysis and minimize bias^2,26^, thus improving the study.

## RESULTS

The results section commonly explains the findings of each variable considered important, which are not always cohesively related to the theory/theoretical model adopted.

Changes in the initial conceptual variables can affect the study’s results, over or underestimating values and relationships according to the theoretical structure^27^. Moreover, a study with no defined theoretical framework could have difficulty determining causal mechanisms, generalizing for other populations, or even establishing the clinical meaning of the effects of the intervention^28^. We therefore consider that presenting the findings for each variable related to the theoretical structure is important, including those in which no statistical relationship or significance was found, those excluded from the initial theoretical model, or even those unreported.

Such results can positively or negatively affect the initial theory/theoretical model or improve it by incorporating new variables, questioning existing variables, or questioning its scientific plausibility. Reporting the study’s innovations to the theory/theoretical model used can expand scientific knowledge and help new research on the subject.

Results can be described using graphic presentations, which show possible changes in the initial theoretical model and the theoretical impacts of the research^29^.

## DISCUSSION

This section opposes ideas and findings of the study with elements of the literature, gathering knowledge to find new propositions. The literature shows that any scientific finding should be assessed regarding the theoretical perspective from which it derives and to which it can contribute^30^, indicating that discussion must be guided by the theoretical and conceptual processes that support the research.

The discussion section should therefore present the observed innovations, the confirmation – or not – of the initial hypothesis, how the chosen theoretical model contributed to the study, and important or needed modifications to the model. Discussing the chosen theory’s limitations is considered as good practice since it shows possible weaknesses of the theoretical approach adopted and of the study itself, helping improve new research.

This form of reporting improves the cohesion and coherence of the discussion, of reflections on the results, and of interpretations and comparisons with other existing theories/models for the same relationships observed.

## CONCLUSION

Once authors understand the importance of the theory/theoretical model for guiding the study, they will instinctively conduct a general overview of the potentialities and gaps of the used model and its implications for future practice and research.

## FINAL CONSIDERATIONS

The study’s theoretical foundation can be seen as its guiding thread. Reporting its specific contribution to each section of the manuscript can improve both the study and science overall.

We understand the challenges related to space limitations imposed by journals. However, available add-in files and/or electronic pages could be a viable alternative to favor robust and transparent reports.

Authors must use theory, methodology, and reporting together to construct scientific knowledge, without overlapping or overvaluing one over the other but understanding their combined importance for scientific doing.

After all, nothing is better than watching a movie or reading a book with a well-founded, well-directed, and well-reported story. We should expect the same from scientific publications. However, are all those involved (authors, reviewers, and editors) concerned with reporting theoretical foundation in scientific articles? Are you?
