# Supplementary material for: Theoretical report: reflections and considerations for authors, reviewers, and editors
Source: Rev Saude Publica. 2022 Apr 13;56:30. doi: 10.11606/s1518-8787.2022056003766 (PMC9018065; doi:10.11606/s1518-8787.2022056003766)
Supplement: Translation

# Translation: Relato teórico: reflexões e considerações para autores, revisores e editores

**Authors:** Breno Augusto Bormann de Souza, Érika Fernandes Tritany, Cláudio José Struchiner

**Affiliations:** I Brasil Escola Nacional de Saúde Pública. Departamento de Epidemiologia em Saúde Pública. Programa de Doutorado em Epidemiologia em Saúde Pública. Rio de Janeiro, RJ, Brasil; II Brasil Universidade de Pernambuco. Escola Superior de Educação Física. Recife, PE, Brasil; III Brasil Universidade Federal do Rio de Janeiro. Faculdade de Medicina. Macaé, RJ, Brasil; IV Brasil Universidade Federal do Rio Grande do Norte. Programa de Doutorado em Saúde Coletiva. Natal, RN, Brasil; V Brasil Fundação Getúlio Vargas. Departamento de Matemática Aplicada. Rio de Janeiro, RJ, Brasil; VI Brasil Universidade Estadual do Rio de Janeiro. Instituto de Medicina Social. Rio de Janeiro, RJ, Brasil

**Keywords:** Estudos Epidemiológicos, Teoria Fundamentada, Medidas, Métodos e Teorias, Publicações

## Abstract

Atualmente, os estudos epidemiológicos voltados à saúde pública têm apresentado limitações importantes quanto ao relato teórico em profundidade, frente a uma supervalorização de aspectos metodológicos. A falta de explicitação teórica nas publicações, afeta não apenas a qualidade do estudo, como também sua reprodutibilidade. Nesse sentido, apoiados em uma revisão da literatura científica acerca do tema, apresentamos neste ensaio reflexões sobre a importância do relato teórico aprofundado, relacionado à fundamentação teórica adotada por pesquisadores nas principais seções do manuscrito (título, resumo, introdução, metodologia, resultados, discussão e conclusão). Acreditamos que este artigo pode favorecer a compreensão sobre a importância e desenvolvimento de relatos teóricos aprofundados em artigos científicos e contribuir para avaliações, interpretações e críticas dos revisores e editores em relação à importância da explicitação e do relato da fundamentação teórica nos manuscritos submetidos aos periódicos científicos.

## INTRODUÇÃO

Teoria e metodologia são consideradas essenciais ao fazer científico por conferirem rigor e qualidade às pesquisas. Entretanto, embora a literatura descreva a importância de sua combinação, esses pilares nem sempre são igualmente relatados^1^. Nesse sentido, falhas de relato (teóricas ou metodológicas) podem reduzir a validade dos achados e impactar negativamente na reprodutibilidade dos estudos^2^.

Em relação ao relato de estudos epidemiológicos, cada vez mais, aspectos metodológicos sobressaem-se aos teóricos. Pressões por produtividade e exigências por publicações em periódicos de alto impacto resultam, muitas vezes, na orientação dos líderes de pesquisa a preferências metodológicas, limitando o universo de estudos a serem desenvolvidos^3^.

Essa atitude é também reforçada por periódicos e a exigência de utilização de instrumentos específicos para medir potenciais vieses metodológicos ou diretrizes de relato desenvolvidas para desenhos específicos (exemplo PRISMA, STROBE, CONSORT), que não aprofundam questões teóricas. Além disso, não são observadas orientações, por periódicos, para o relato da fundamentação teórica; e evidencia-se escassez de instrumentos que auxiliem autores ao relato teórico aprofundado.

Todos os estudos devem estar ligados a uma teoria e/ou modelo teórico que baseie e guie as etapas da pesquisa^4^, cujo relato aprofundado facilite sua identificação e oriente tanto a equipe de pesquisa quanto o leitor. A falta de citação e explicitação de teorias ou modelos teóricos nos estudos epidemiológicos pode prejudicar a compreensão, limitar a avaliação crítica, comprometer a qualidade do estudo e a reprodutibilidade^2,5,6^, uma vez que a reprodutibilidade não depende exclusivamente da qualidade metodológica, mas da combinação entre qualidades metodológica, teórica e de relato^2^.

Para Cabrera^1^, a comunicação científica rigorosa está atrelada à citação da teoria adotada; descrição de suas variáveis e construtos; explicitação de como a fundamentação teórica orientou a metodologia e suas análises; e, por fim, com base nos componentes explicativos da teoria ou descrições dos modelos teóricos, a discussão das conclusões ou questões temáticas abordadas. Portanto, o intuito do relato teórico não está centrado na repetição mecânica e exaustiva do referencial teórico, mas na compreensão de sua importância em todas as etapas da pesquisa e do manuscrito.

Assim, não basta citar em cada parte do texto uma frase que remeta à teoria, como “…este estudo foi baseado na teoria/modelo teórico x”, mas explicitar suas contribuições para cada seção do artigo, de forma coerente com sua função no texto, bem como, reciprocamente, possíveis impactos do estudo à Teoria/Modelo. Nesse sentido, apresentamos reflexões e considerações sobre a importância do relato teórico aprofundado em cada seção de um artigo científico.

## TÍTULO

Sumariamente o título deve refletir a essência do manuscrito, sua novidade e relevância para a ciência^7^, não havendo, entretanto, consenso sobre um tamanho ideal^8,9^. Indica-se que resuma a ideia principal do manuscrito de maneira simples e com estilo, identificando variáveis-chave, teorias adotadas e a relação entre elas^10^, sendo suficientemente informativo, descritivo e preciso a ponto de despertar interesse e favorecer sua identificação^11^.

No que tange à elaboração de revisões e buscas bibliográficas sobre teorias e sua utilização, observa-se que a ausência de relato da teoria/modelo teórico no título dificulta e pode impossibilitar a seleção em completude de artigos que as utilizem. A leitura dos títulos é comumente sugerida como etapa inicial de busca e seleção bibliográfica. Assim, a ausência da teoria no título pode resultar em lacunas nos achados bibliográficos e diminuição do alcance das revisões^12^.

Portanto, ganha relevância sua explicitação no título, facilitando a identificação do artigo, e apresentando previamente aos leitores o enfoque teórico adotado. Afinal, determinar o fator que torna seu artigo único e destacável no campo é um ponto importante e sua ênfase no título pode estimular o interesse e seu potencial acesso^11^.

## RESUMO

A seção resumo objetiva auxiliar os leitores a selecionar artigos apropriados mais rapidamente; permitir pesquisas mais precisas; e facilitar a revisão por pares^13^. Podem ser “estruturados” ou “não estruturados”, sendo que o conteúdo pode ser o mesmo, diferenciando-se pelo formato de apresentação^14^. Entretanto, o resumo estruturado tem se mostrado mais efetivo e sistemático^15^, o que impele muitos periódicos a recomendar sua adoção.

Esse formato fornece aos revisores um sentido imediato e geral do estudo, auxiliando na estruturação da análise e revisão do artigo^15^. Entretanto, embora apresente vantagens^13^, são apontadas quantidades substanciais de informações ausentes nos resumos, ainda que presentes no texto^16^, principalmente aquelas relacionadas à fundamentação teórica e sua contribuição nas seções do manuscrito^17^.

É importante que informações preliminares sobre o quadro teórico sejam apresentadas no resumo, com a explicitação de como as variáveis foram avaliadas, os principais achados, potenciais limitações e conclusões relacionadas à teoria/modelo teórico adotado, ainda que de modo simplificado^10,18^. Trata-se da sintética explanação da metodologia utilizada, resultados obtidos, limitações identificadas e conclusões – seções já comumente relatadas nos estudos e resumos –, levando-se em consideração os desafios e potencialidades da teoria/modelo utilizado, sua contribuição no estudo e como a pesquisa pôde contribuir para seu fortalecimento ou questionamento. Essa prática fornece um relato coerente e coeso, e pode potencializar a seleção, acesso e avaliação do artigo.

## INTRODUÇÃO

Defende-se que a introdução inicie uma breve explanação do assunto, conforme a literatura e fundamentação teórica adotada^19^. O conhecimento atual, *insights* e desenvolvimentos recentes sobre o tema e possíveis lacunas devem ser apresentados, justificando as escolhas teóricas e metodológicas^20^. Nessa seção, os autores encontram maior liberdade para contextualização e fundamentação do estudo de forma mais ampla e descritiva.

Entretanto, essa seção é comumente apresentada de forma sucinta com o simples intuito de convencer o leitor do valor do seu produto^21^. Essa atitude – conhecida popularmente como “vender o peixe” –, muitas vezes associada à supervalorização de aspectos metodológicos, pode derivar de pressões por aumento da produtividade científica, ainda que em detrimento de sua qualidade, o que deve ser combatido^22,23^.

A introdução deve apresentar a base que sustenta as escolhas teórico metodológicas desenvolvidas e as questões a serem respondidas ao longo do texto, com explicitação e descrição da fundamentação teórica, para situar o leitor sobre o referencial utilizado; sua valorização científica ou críticas; a existência de teorias paralelas ou lacunas na literatura; e motivos que orientaram sua escolha, com base em hipóteses sobre o que a teoria/modelo teórico adotado pode incorporar de novo ao fenômeno estudado.

Outrossim, não apenas teorias consagradas e grandes cânones da literatura estariam passíveis a essa apresentação inicial^5^. Sabemos que muitos pesquisadores apresentam inovações em termos de relações causais e, para isso, frente à escassez de literatura que apoie suas hipóteses, criam modelos teóricos com base na livre conexão entre diferentes temas e/ou achados científicos prévios. Nesse sentido, faz-se necessária a explanação do modelo teórico criado; suas variáveis e motivos de escolha ou exclusão; suas inter-relações; e possibilidades de defesa do modelo teórico adotado como uma fonte de inovação para a temática.

## METODOLOGIA

Além de todo conhecimento ser carregado de teoria, todos os métodos são guiados por teorias^24^. Apesar da seção metodologia ser comumente desenvolvida apenas para apresentação dos métodos de investigação/intervenção utilizados, torna-se importante o aprofundamento do relato da fundamentação teórica que subsidia o caminho metodológico.

Explicitar como a teoria/modelo teórico orientou o método; como as variáveis foram classificadas e inter-relacionadas; justificar a exclusão e inclusão das variáveis nas análises e a presença de vieses nada mais é do que a profunda explanação do caminho teórico-metodológico adotado. Não se trata apenas da citação do método escolhido – desconectado do arcabouço teórico e das evidências sobre o tema –, mas a justificativa, em profundidade, de cada ponto de importância para as análises realizadas e sua correspondência ou não com a fundamentação teórica.

Defendemos que a escolha das variáveis seja orientada pela teoria/modelo teórico (existente, adaptado ou novo, criado pelos pesquisadores) e não por decisões arbitrárias como facilidade/dificuldade de obtenção dos dados ou mesmo preferências dos pesquisadores, devendo ser justificada a impossibilidade de obtenção de informações de variável(is) considerada(s) importante(s).

Além disso, uma apresentação gráfica do modelo teórico faz-se necessária, pois promove maior clareza sobre as variáveis envolvidas e suas relações^2^. Todo modelo teórico é uma simplificação da realidade e sua representação gráfica não seria diferente^25^. Entretanto, em meio ao grande volume textual produzido, descrições detalhadas dos fenômenos, críticas e considerações, pode ser difícil para pesquisadores e leitores visualizar o modelo teórico escolhido^6^.

A representação gráfica do modelo teórico apresenta papel importante na comunicação da pesquisa: facilita a visualização das variáveis de interesse e suas interrelações; promove aprendizagem significativa do fenômeno estudado^2^; possibilita o aprimoramento contínuo do modelo teórico pela revisão das variáveis e observação de relações não consideradas inicialmente; auxilia na construção do modelo de análise e minimização de vieses^2,26^. Ou seja, contribui para o aprimoramento do estudo.

## RESULTADOS

Na seção resultados já é prática corrente a explicitação dos achados de cada variável considerada importante, porém nem sempre se apresentam relacionadas de forma coesa à teoria/modelo teórico adotado.

Alterações nas variáveis conceituais iniciais podem gerar modificações nos resultados do estudo, supra ou subestimando valores e relações de acordo com a estrutura teórica^27^. Além disso, um estudo sem um arcabouço teórico definido pode dificultar a determinação de mecanismos causais, generalização para outras populações ou, ainda, o estabelecimento do significado clínico dos efeitos da intervenção^28^. Dessa forma, julgamos importante a apresentação dos achados finais encontrados para cada variável relacionada à estrutura teórica, inclusive aquelas em que não foi encontrado relação ou significância do ponto de vista estatístico, excluídas do modelo teórico inicial ou mesmo não relatadas.

Tais resultados podem impactar positiva ou negativamente na teoria/modelo teórico inicial, possibilitando seu aprimoramento (seja pela incorporação de novas variáveis, pelo questionamento de variáveis existentes ou pelo questionamento de sua própria plausibilidade científica). Dessa forma, relatar as inovações trazidas por seu estudo à teoria/modelo teórico utilizado pode contribuir com a construção do conhecimento científico e auxiliar novas pesquisas sobre o tema.

A descrição dos resultados pode ser acompanhada de apresentações gráficas que demonstrem as modificações realizadas no modelo teórico inicial, quando houver, permitindo a observação dos impactos teóricos gerados pela pesquisa^29^.

## DISCUSSÃO

Nessa seção ocorre a contraposição de ideias e achados do estudo com elementos da literatura, realizando uma conjunção de saberes que permite novas proposições. A literatura evidencia que qualquer achado científico deve ser avaliado em relação à perspectiva teórica da qual deriva e para a qual possa contribuir^30^, o que fortalece a necessidade de orientação da discussão com base nos processos teóricos e conceituais que fundamentam a pesquisa.

Dessa forma, é importante a apresentação das inovações observadas, a confirmação – ou não – da hipótese inicial, e o modo como o modelo teórico escolhido contribuiu para o estudo e possíveis modificações importantes ou necessárias ao modelo teórico, frente aos resultados. Discutir também limitações inerentes à teoria escolhida é visto como boa prática no sentido de explicitar possíveis fragilidades do enfoque teórico adotado e do estudo em questão, bem como fomentar o aprimoramento de novas pesquisas.

Essa forma de relato facilita a coesão e coerência da discussão e reflexões sobre os resultados, bem como interpretações e comparações com outras teorias/modelos existentes para as mesmas relações observadas.

## CONCLUSÃO

Uma vez compreendida a importância da teoria/modelo teórico na orientação do estudo, um apanhado geral sobre potencialidades e lacunas do modelo teórico utilizado, bem como suas implicações para a prática e pesquisas futuras será uma tarefa instintiva aos autores.

## CONSIDERAÇÕES FINAIS

A fundamentação teórica que baseia o estudo pode ser vista como um fio condutor do trabalho, e o relato de sua contribuição específica para cada seção do manuscrito pode aprimorar não apenas o estudo, mas a ciência como um todo.

Compreendemos os desafios relativos às limitações de espaço pelos periódicos. Entretanto, a disponibilização de arquivos de suplemento e/ou páginas eletrônicas pode ser uma alternativa viável para favorecer relatos robustos e transparentes.

Teoria, metodologia e relato devem caminhar juntas na construção do conhecimento científico, sem que exista sobreposição ou supervalorização de uma em detrimento da outra, compreendendo sua importância combinada para o fazer científico.

Afinal, nada melhor que assistir a um filme ou ler um livro que apresente uma história bem fundamentada, dirigida e relatada. Devemos esperar o mesmo das publicações científicas. Entretanto, será que todos os envolvidos (autores, revisores e editores) estão se preocupando com a fundamentação teórica e seu relato nos artigos científicos? E você?
